# Human alveolar macrophage metabolism is compromised during *Mycobacterium tuberculosis* infection

**DOI:** 10.3389/fimmu.2022.1044592

**Published:** 2023-01-26

**Authors:** Laura E. Mendonca, Erwan Pernet, Nargis Khan, Joaquin Sanz, Eva Kaufmann, Jeffrey Downey, Alexandre Grant, Marianna Orlova, Erwin Schurr, Connie Krawczyk, Russell G. Jones, Luis B. Barreiro, Maziar Divangahi

**Affiliations:** ^1^ The Research Institute of the McGill University Health Centre, Meakins-Christie Laboratories, Department of Medicine, Department of Microbiology and Immunology, Department of Pathology and; ^2^ McGill International TB Centre, Montreal, QC, Canada; ^3^ Institute for Biocomputation and Physics of Complex Systems (BIFI) for Biocomputation and Physics of Complex Systems and Department of Theoretical Physics, University of Zaragoza, Zaragoza, Spain; ^4^ Department of Medicine and Human Genetics, McGill University. Program in Infectious Diseases and Immunity in Global Health, The Research Institute of the McGill University Health Centre, Montreal, QC, Canada; ^5^ Department of Physiology, Goodman Cancer Research Centre, McGill University, Montreal, QC, Canada; ^6^ VanAndel Institute, Center for Cancer and Cell Biology, Grand Rapids, MI, United States; ^7^ Department of Genetics, Centre hospitalier de l'Université (CHU) Sainte-Justine Research Center, Montreal, QC, Canada; ^8^ University of Chicago, Department of Medicine, Section of Genetic Medicine, Chicago, IL, United States

**Keywords:** tuberculosis, immunometabolism, immunity, pulmonary macrophages, metabolic reprograming, cell death programs

## Abstract

Pulmonary macrophages have two distinct ontogenies: long-lived embryonically-seeded alveolar macrophages (AM) and bone marrow-derived macrophages (BMDM). Here, we show that after infection with a virulent strain of *Mycobacterium tuberculosis* (H37Rv), primary murine AM exhibit a unique transcriptomic signature characterized by metabolic reprogramming distinct from conventional BMDM. In contrast to BMDM, AM failed to shift from oxidative phosphorylation (OXPHOS) to glycolysis and consequently were unable to control infection with an avirulent strain (H37Ra). Importantly, healthy human AM infected with H37Ra equally demonstrated diminished energetics, recapitulating our observation in the murine model system. However, the results from seahorse showed that the shift towards glycolysis in both AM and BMDM was inhibited by H37Rv. We further demonstrated that pharmacological (e.g. metformin or the iron chelator desferrioxamine) reprogramming of AM towards glycolysis reduced necrosis and enhanced AM capacity to control H37Rv growth. Together, our results indicate that the unique bioenergetics of AM renders these cells a perfect target for *Mtb* survival and that metabolic reprogramming may be a viable host targeted therapy against TB.

## Introduction

*Mycobacterium tuberculosis* (*Mtb)* is transmitted *via* the aerosol route and replicates mainly in pulmonary macrophages. The success of *Mtb* is largely dependent on these initial events. However, few studies have aimed to characterize the initial events following *Mtb* infection.

Macrophages exist in virtually all tissues and considerable studies highlight the heterogeneity of tissue macrophage origins as well as their microanatomical niche that regulate their functions (1). Alveolar macrophages (AM) are a unique macrophage subtype in the airways and are seeded in the lungs during embryogenesis from fetal liver monocytes and are maintained by self-renewal with minimal contribution from bone marrow circulating monocytes (2, 3). Moreover, considerable heterogeneity also exists within the recruited monocyte-derived pool, with cells exhibiting a spectrum of “classical” inflammatory (termed M1) or “alternative” repair (termed M2) response profiles, suggesting substantial effector plasticity within the recruited compartment (4) with almost unknown effects on control of infection. We have demonstrated that systemic BCG vaccination reprograms hematopoietic stem cells (HSCs) to generate a unique population of BMDM (trained macrophages) that can migrate to the lung providing protection against *Mtb* (5). Similarly, studies in NHP have also demonstrated that systemic BCG vaccination provide superior protection or even prevention against TB (6–9). In contrast to BCG, we have also recently shown that virulent *Mtb* accesses the bone marrow to prevent trained immunity in macrophages in RD1-dependent manner (10). Thus, *Mtb* has evolved sophisticated mechanisms to subvert early inflammatory responses in both alveolar and recruited macrophages.

Accumulating evidence indicates AM as the main replicative niche of *Mtb* due to impaired antimycobacterial responses. Although the exact mechanism(s) for the impaired immunity of AM to *Mtb* is incompletely understood, a recent study has suggested that the Nuclear factor erythroid 2-related factor 2 (Nrf2)-dependent antioxidant pathway dominates early AM responses to *Mtb*, rendering them less bactericidal (11). Moreover, studies have linked glycolysis with mycobacterial control in mice (12, 13) and the glycolytic pathway is impaired in AM (14). Additionally, while it has been shown that human macrophages exhibit a metabolic switch to glycolysis when stimulated with irradiated non-replicating *Mtb* (15) recent findings indicate that macrophage glycolysis is inhibited following infection with *viable Mtb* due to induction of microRNA miR-21 (13). These data indicate that *Mtb* reprograms macrophage metabolic responses to its own advantage.

In the current study, we investigate the limitations of the AM metabolic program as compared to a spectrum of macrophages derived from BM precursors in providing optimal host defense against viable *Mtb*. We used an *in vitro* system to specifically model the metabolism of both murine and human AM versus BMDM (both M1 and M2) or MDM and we recapitulated the *in vivo* setting showing that AM are more permissive than BMDM to *Mtb* infection. AM and BMDM transcriptomic analysis identified metabolic pathways that significantly differed under steady state and after infection with *Mtb*. Subsequently, by using the Seahorse assay, we found that AM but not BMDM showed enhanced oxidative phosphorylation (OXPHOS) compared to glycolytic metabolism and consequently were unable to control H37Ra infection. We then extended our observations to human macrophages, providing the first demonstration that in sharp contrast to monocyte-derived macrophages (MDM), AM from healthy subjects were unable to energetically step-up after H37Ra infection. However, in contrast to H37Ra, H37Rv inhibits glycolytic pathways in both AM and BMDM indicating virulence factors of H37Rv further target macrophage metabolism. Finally, we demonstrate that AM metabolism can be reprogrammed using iron chelators or metformin that inhibit necrosis and increase their antimycobacterial activity. This result is consistent with previous studies demonstrating how virulent strains of *Mtb* induce necrosis in macrophages to promote TB pathogenesis (16, 17). Thus, AM are metabolically impaired to control *Mtb* growth, while modulation of AM metabolism (e.g. shifting to glycolysis) or recruited macrophages (e.g. BCG IV vaccination) (5), may provide a novel avenue in treatment against TB.

## Methods

### Cell culture

For murine bone marrow-derived macrophages (BMDM), femurs and tibias from six- to ten- week old C57BL/6 mice (bred in-house, approved by the Animal Research Ethics Board of McGill University, project ID: 5860) were harvested aseptically. Bones were flushed with R10% (RPMI-1640 supplemented with 10% heat-inactivated fetal bovine serum (FBS), both from Wisent), and whole bone marrow was plated in 9 mL BMDM medium (RPMI-1640 supplemented with 10% heat-inactivated FBS, 2% HEPES, 1mM sodium pyruvate, 1% essential amino acids, 1% non-essential amino acids and 100U/mL penicillin/streptomycin, all from Wisent) containing 30% L929 cell- (ATCC) conditioned medium at a density of 5-10x10^6^ cells per non-TC-treated petri dish and cultured at 37°C, 5% CO_2_. After 3 days, 9 mL of fresh BMDM medium containing 30% L929 media was added to each dish. On day 6, cells were washed with PBS to remove non-adherent cells and detached with cell stripper (Corning).

After differentiation, BMDM were plated at 1x10^6^/well in a 6-well plate or 1x10^5^/well in a Seahorse XF96 assay plate in BMDM medium and allowed to adhere for 24 hours prior to stimulation with 100 ng/mL LPS and 20 ng/mL IFNγ to produce M1-like macrophages or 20 ng/mL IL-4 to produce M2-like macrophages. Macrophages were stimulated for 24 hours prior to further use.

To generate human PBMC-derived macrophages (MDM), peripheral blood mononuclear cells (PBMCs) were isolated from healthy donors using Lymphocyte Separation Medium (Wisent), according to the manufacturer’s protocol. PBMCs were then cultured in 10mL RPMI with 10% FBS with 50ng/mL of recombinant human M-CSF (Peprotech) in non-TC-treated petri dishes. Monocytes were differentiated for 7 days with fresh media (10mL) added after 3 days of culture. After 7 days of culture, half culture medium was removed and replaced by fresh media containing 50ng/mL of human M-CSF. After 3 days, fresh media with human M-CSF was added. After another 3 days, cells were washed with PBS to remove non-adherent cells and detached with cell stripper (Corning). Cells were seeded at 100,000/well in R10% and used for further experiments.

### Alveolar macrophages

For murine alveolar macrophages (AM), AM were harvested and pooled from the bronchoalveolar lavage (BAL) of six- to ten- week old C57BL/6 mice. Briefly, mice were euthanized with CO_2_ and the intact lung and trachea complex was carefully removed. A cannula was inserted into the trachea and firmly secured using surgical string. Lungs were then lavaged *ex vivo* five times with 1 mL of ice cold sterile PBS while gently massaging the tissue to maximize recovery. Purity was greater than 95% as assessed by flow cytometry, and non-adherent cells were washed away 4h after plating in BMDM medium.

Human AM were obtained from healthy volunteers who underwent BAL in the Centre for Innovative Medicine at the MUHC, in collaboration with Dr. Erwin Schurr. The study is approved by the REB of the MUHC (study number MP-CUSM-15-406). To obtain AM, the BAL cells were spun down at 1200 rpm for 10 minutes and washed once with RPMI supplemented with 2% human serum (Wisent), 1% penicillin/streptomycin, 2.5 ug/ml of Amphoterecin B (Wisent) and further resuspended in R10%. Cells were seeded at 100,000/well and used for further experiments.

### *In vitro* macrophage infection

Virulent *Mycobacterium tuberculosis* strain H37Rv and avirulent strain H37Ra were grown in a liquid culture of Middlebrook 7H9 medium (Difco) containing 0.2% glycerol (Fisher), 0.05% Tween-80 (Sigma-Aldrich) and 10% albumin-dextrose-catalase. Bacteria were grown to log phase, as determined by an optical density of 0.3 to 0.8, prior to infection. For H37Rv, cells were infected at a multiplicity of infection (MOI) of 1, whereas for H37Ra macrophages were infected at an MOI of 2.5. Macrophages were cultured for at least 4 hours in BMDM medium without Pen/Strep prior to infection. 4 hours post-infection, macrophages were washed 3 times with R10% and cultured in 1 mL BMDM medium without Pen/Strep. At 4h, 24h, 48h and 72h post-infection, cells and supernatants were harvested and centrifuged for 5 minutes at 15,000 rpm in a microcentrifuge to pellet down the cells and bacteria. Supernatant was then collected and filtered for downstream assays. The cell pellet was resuspended in 500 µL of sterile water and lysed by incubating for 5 minutes at room temperature. Following cell lysis, 500 µL of PBS-Tween-80 (0.05%) was added. Colony-forming units (CFUs) were determined by plating serial dilutions of lysates in PBS-Tween-80 (0.05% Tween-80) on Middlebrook 7H10 agar (Difco) plates containing 0.5% glycerol (Fisher), 10% oleic acid-albumin-dextrose-catalase and PANTA (BD Biosciences). Colonies were counted 3 weeks post-plating. Transcriptomics and metabolic analyses were performed 24 hours post-*Mtb* infection based on previous published papers from our and other groups (5, 10, 15, 18). More specifically for this study, we observed that following *in vitro* macrophages infection, the bacterial loads were significantly higher in AM at 48 hours post-infection. Therefore, we analyzed at 24-hour post-*Mtb* infection to make sure that the number of bacteria had no impact on transcriptomic profile and cellular metabolism of the different macrophage populations. In some experiments, AM were treated with Metformin (100 and 200µM, Sigma) or Desferrioxamine (100µM, Sigma) for 24 hours before infection with *Mtb*.

In some experiments Mtb *H37Rv* was grown in presence of 100 or 200µM of desferrioxamine (DFX) or Metformin.

### Extracellular flux analysis

Real-time oxygen consumption rates (OCR) and extracellular acidification rates (ECAR) of macrophages were measured in XF media (non-buffered DMEM containing 2mM L-glutamine, 25mM glucose and 1mM sodium pyruvate) using a Seahorse XFe 96 Analyzer (Agilent Technologies). For the mitochondrial stress test, mitochondrial inhibitors oligomycin (1µM, Sigma), fluorocarbonyl cyanide phenylhydrazone (FCCP, 1.5µM, Sigma), antimycin A (0.5µM, Sigma) and rotenone (0.5µM, Sigma) were used. Briefly, macrophages were seeded at a density of 100,000 cells per well and 3 basal measurements were taken. Following this, 2 consecutive measurements were taken following each injection of oligomycin, FCCP, and antimycin A with rotenone. Basal respiration was determined as the last basal measurement minus the non-mitochondrial respiration rate, as determined by the last reading following antimycin A/rotenone injection. ATP production was determined as the decrease in OCR following oligomycin injection and coupling efficiency is determined by the ratio of ATP production to basal respiration. Finally, spare respiratory capacity was determined as the absolute increase in OCR after FCCP injection compared to basal respiration. All measurements were normalized to cell number using a crystal violet dye extraction assay. Oxygen consumption curves were generated using Wave Desktop 2.3 (Agilent Technologies).

### Cytokine production

The production of IL-6, IL-12p40, TNF-α, and IL-10 was measured using commercial enzyme-linked immunosorbent assay (ELISA) kits (R&D systems) according to the manufacturer’s instructions.

#### Cell death assay

Ferroptosis, apoptosis and necrosis were assessed in AM and BMDM 24 hours post-infection with H37Rv (MOI=10), using MDA lipid peroxidation (Abcam), Cell death ELISA (Sigma) and LDH assay (Promega), respectively.

### Flow cytometry

For BMDM staining, macrophages were harvested from 6-well plates using 1mL of Cell Stripper (Corning). Cells were then washed and incubated for 10 minutes with anti-CD16/32 in FACS buffer (0.5% BSA/PBS) at 4°C to block non-specific antibody interaction with Fc receptors. They were then stained for 30 minutes at 4°C with the following antibodies (ebioscience or BD unless otherwise stated): anti-CD11b PE-Cy7, anti-F4/80 APC-efluor780, anti-CD86 V450, anti-CD206 APC (Biolegend). All samples were fixed in 1% paraformaldehyde prior to acquisition using a BD LSRFortessa X-20 flow cytometer (BD Biosciences) and FACSDiva software (BD Biosciences). Data analysis was performed using FlowJo v.10 (TreeStar).

In some experiments, AM and BMDM were stained with Calcein-AM (Invitrogen) or mitochondrial iron-specific dye RPA (Biotium) following manufacturer’s instructions to measure the cellular iron labile pool and mitochondrial iron levels, respectively. In these experiments, the Δ geometric mean fluorescence intensity (ΔgMFI) is shown by substracting the gMFI values of unstained from stained samples. For both compounds, the fluorescence is quenched in presence of iron and inversely correlated to iron levels. Thus, the lower the Calcein and RPA fluorescence intensity, the higher the levels of cytosolic and mitochondrial iron, respectively.

#### Murine RNAseq analyses

RNA was extracted using Trizol (Invitrogen) from samples of AM, M1 and M2 macrophages (AM, n= 3 biological replicates/group; BMDM, n=4 biological replicates/group) infected or not with H37Rv (MOI=1, 24 hours). First, adaptor sequences and low-quality score bases (Phred score < 20) were trimmed using Trim Galore (version 0.2.7). The resulting reads were mapped to the mouse genome reference sequence (Ensembl GRCm38 release 81) using kallisto (v0.43.0) (19). Then, protein coding genes were selected, and samples were normalized using the weighted trimmed mean of M-values algorithm (TMM), as implemented in the R package edgeR (20). Then, reads were log2-transformed using voom, within the limma package (21), and low-expressed genes, defined as those showing median log2-transformed expression lower than 2 within all conditions (i.e. combination of cell-type and infection status) were filtered out. This resulted in a reads matrix of 10401 genes.

#### Differential expression analyses

The filtered matrix was then re-normalized, using Edger and voom, and modelled according to the following paired and nested design, using limma (21):

Expression~ Mouse3+3Macrophage_type + H37RV_infection: Macrophage_type

From the mentioned model design, we retrieved the following Differential expression contrasts across cell-types, within infection status:

- M2 - AM at NI
- M1 - AM at NI
- M2 – M1 at NI.
- M2 - AM at RV
- M1 - AM at RV
- M2 – M1 at RV

As well as the following infection-DE contrasts within macrophage type:

- RV - NI at AM.
- RV - NI at M1
- RV - NI at M2

And, finally, the contrast corresponding to differential responses to infection between AM and either M1 or M2:

- (RV - NI at M1) – (RV - NI at AM)
- (RV - NI at M2) – (RV - NI at AM)
- (RV - NI at M2) – (RV - NI at M1)

Finally, we corrected for multiple testing using Benjamini-Hochberg false discovery rates. The results of these analyses are reported in the **Supplementary Table S1**.

#### GO enrichments and visualization

Gene ontology enrichment analyses were conducted using the web-tool GOrilla (22), using the list of human GO annotations, and the one, sorted gene-list setup (**Supplementary Table S2**), for which, ordered standardized effect sizes (i.e. t-statistics) were used as an input.

**Figure 1:**
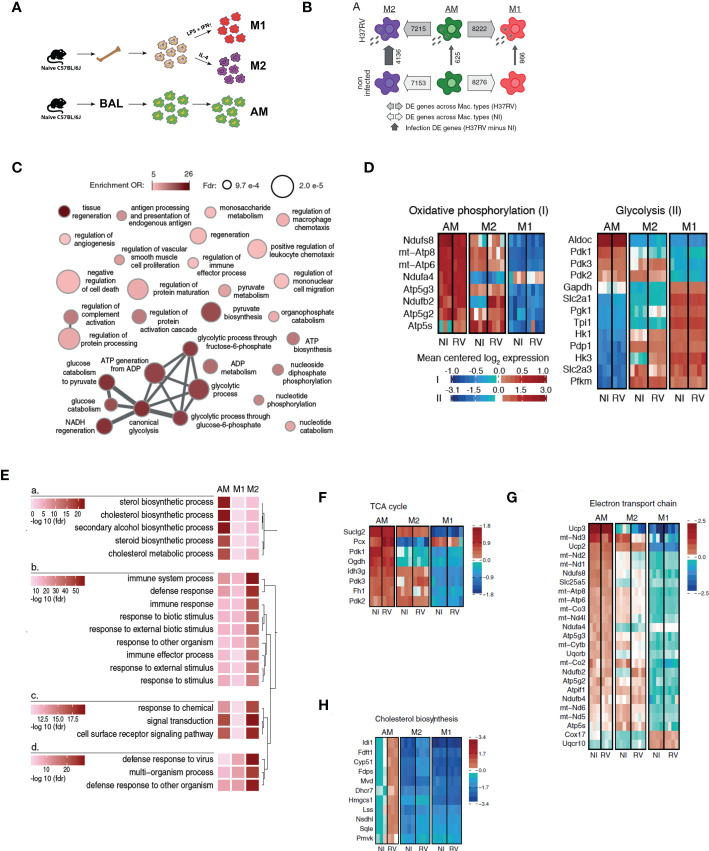
Alveolar macrophages have a highly divergent transcriptomic profile with respect to either M1 or M2 macrophages. **(A)** Bone marrow was harvested from naïve C57BL/6J mice and cultured in the presence of L929-conditioned media for 6 days prior to polarization with either LPS and IFNγ (M1), or IL-4 (M2) for 24hrs. Alternatively, alveolar macrophages (AM) were harvested from BAL of naïve mice. Cells were infected with *Mtb* H37Rv (MOI=1) for 24 hours. (AM n=3 biological replicates, M1/M2 BMDM n=4 biological replicates). **(B)** Numbers of differentially expressed genes between macrophage types and infection states (at 5% FDR, see also **Table S1**). **(C)** Network visualization of the GO terms most significantly enriched among genes showing a significantly lower expression in AM than in M1 non-infected macrophages (see also **Table S2**). Node size is proportional to its statistical significance (all nodes show an FDR<1x10^-3^), while color illustrates enrichment’s effect size (OR>5) and connections indicate semantic similarity between terms (i.e. genes shared). **(D)** Expression heatmaps for genes annotated within the metabolic pathways “Oxidative phosphorylation” and “glycolysis” in www.wikipathways.org. Only genes showing most significant differential expression changes with respect to any of the contrasts presented in **Table S1** were plotted (FDR < 0.05 and abs(logFC)>1). **(E)** Comparative enrichment results for the GO terms most significantly enriched among genes up-regulated upon H37RV infection in the three types of macrophages. **(F–H)** Expression heatmaps for genes annotated within the metabolic pathways “TCA cycle” **(F)**, “Electron transport chain” **(G)** and “Cholesterol biosynthesis” **(H)** in www.wikipathways.org. Only genes showing most significant differential expression changes with respect to any of the contrasts presented in **Table S1** were plotted (FDR < 0.05 and abs(logFC)>1).

From the output of the analyses corresponding to lower expression in AM with respect to M1 non-infected macrophages, we selected the most strongly enriched terms (OR>5 and FDR<0.001), and used those to produce network visualizations using REVIGO (23) (graph size: medium, semantic similarity measure: Jiang and Conrath), and cytoscape (24), which we show in **Figure 1B**. Finally, to compare the most enriched terms among genes up-regulated upon infection in each of the macrophage types, we selected the union of GO terms in the top 10 most significant enrichments in each case, to obtain the set of 20 terms represented in **Figure 1D**, which we then structured through hierarchical clustering.

### Statistical analysis

Statistical analysis was performed using GraphPad Prism version 7.02 (GraphPad Software, San Diego, California, USA). All data are presented as mean ± SEM and differences were considered significant if p<0.05. 1-way ANOVA followed by Tukey comparison or 2-way ANOVA followed by either Bonferroni, Sidak’s or Dunnetts comparison was used, as specified in each figure.

## Results

### Transcriptomic profiling of AM vs BMDM

**Figure 2:**
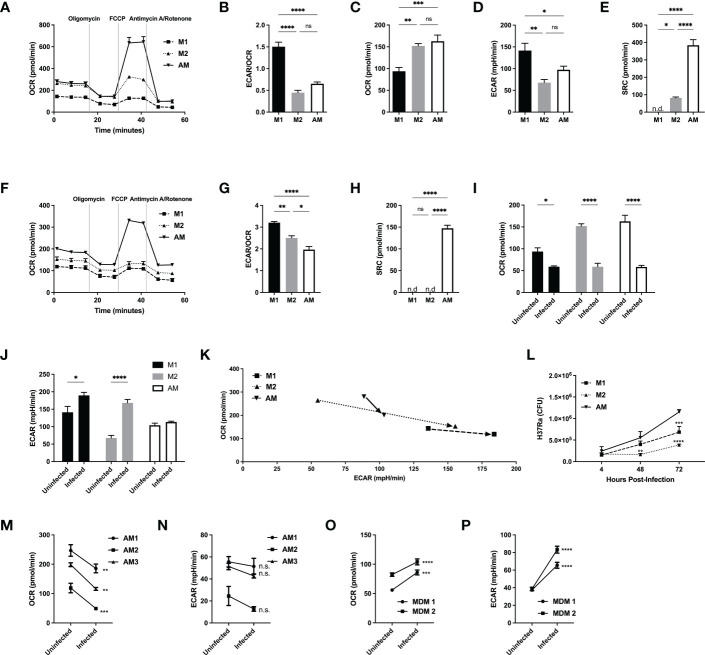
Alveolar macrophages have a unique metabolic signature, distinct from M2 macrophages and do not switch to a glycolytic phenotype following avirulent (H37Ra) *Mtb* infection. M1 and M2 bone marrow-derived and naïve alveolar macrophages were subjected to a Seahorse assay to measure cellular metabolism before **(A–E)** and after 24hrs of infection **(F–K)** with H37Ra at MOI of 2.5. **(A)** Oxygen consumption rate (OCR) curve following sequential injections of mitochondrial inhibitors oligomycin, FCCP and antimycin/rotenone (Ant Rot). **(B)** Ratio of extracellular acidification rate (ECAR)/OCR. **(C)** Quantification of basal levels of oxygen consumption, **(D)** ECAR, **(E)** spare respiratory capacity (SRC). **(F)** Oxygen consumption rate (OCR) curve of infected macrophages following sequential injections of mitochondrial inhibitors oligomycin, FCCP and antimycin/rotenone. **(G)** Ratio of ECAR/OCR of infected macrophages. **(H)** Quantification of spare respiratory capacity of infected macrophages. Shift in **(I)** OCR and **(J)** ECAR following infection, illustrated in **(K)**. **(L)** M1 and M2 bone marrow-derived and naïve alveolar macrophages were infected at MOI 2.5 with H37Ra and subjected to CFU assays. **(M–P)** Human alveolar macrophages and PBMC-derived MDMs were subjected to a Seahorse assay to measure cellular metabolism before and after 24hrs of infection with H37Ra at a MOI of 2.5. Oxygen consumption rate (OCR) shift of hAM **(M)** and MDM **(O)** prior and after infection. Extracellular acidification rate shift of hAM **(N)** and MDM **(P)** prior and after infection. Data are given as mean ± SEM. The results are representative of 2 independent experiments **(A–L)** and 3 **(M, N)** or 2 **(O, P)** independent healthy donors. * p<0.05; ** p<0.01; *** p<0.001; **** p<0.0001 (1-way ANOVA followed by Tukey comparison) **(D, E, M–P)** 2-way ANOVA followed by Bonferroni comparison or Sidak’s test.

We initially established an *in vitro* M1/M2 model to investigate how AM and BMDM would be placed within these two extremities of the macrophage spectrum and we speculated that M1 are representative of recruited macrophages and M2 are representative of AM. We generated M1, M2 and AM were collected from bronchoalveolar lavage (BAL) of naïve mice **(** **Figure 2A** **)**. By flow cytometry, M2 showed significant upregulation of CD206 when compared to AM or M1 while M1 showed significant upregulation of CD86 **(** **Supplementary Figures S1A, B** **)**. Surprisingly, AM did not resemble to either M1 or M2 in terms of expression of these surface markers, supporting the recent hypothesis that AM reflect neither M1 nor M2-like macrophages (25).

Macrophage phenotype is highly dependent on their microenvironment and regulated at both transcriptional and metabolic levels (26). We characterized the transcriptional signature associated with AM, M1, and M2 at baseline and after *Mtb* (H37Rv) infection by RNA-Seq. In agreement with our flow cytometry analyses, the AM transcriptional profile was markedly different from both M1 and M2. At a false discovery rate (FDR) of 5%, 8,276 and 7,153 genes were differentially expressed in AM in comparison to M1 and M2, respectively (79.6% and 68.8% of all genes tested; **Figure 2B**, **Supplementary Table S1**). Gene ontology enrichment analyses revealed that, genes that were more highly expressed in M1 than AM were strongly enriched in inflammatory responses (OR=2.68, FDR=6.35x10^-6^; **Supplementary Table S2**). Importantly, we also found marked differences in gene expression involved in key metabolic pathways. Specifically, M1 showed a strong up-regulation of genes involved in glycolytic process (OR=19.25, FDR=4.31x10^-5^) and pyruvate metabolism (OR=8.74, FDR=4.8x10^-4^) while AM expressed genes predominantly involved in OXPHOS (**Figure 2C**). To further study variation in metabolic pathways between AM, M1 and M2, we extended our analyses using gene sets annotated within the metabolic pathways glycolysis and OXPHOS and whose expression levels vary most strongly across macrophage types and/or infection states (differently expressed genes at FDR < 0.05 and abs(logFC)>1 in any of the DE contrasts presented in **Supplementary Table S1**).

AM displayed unique metabolic signatures compared to M1 (**Figures 2C–G**). M1 showed a characteristic glycolytic signature marked by increased expression of genes involved in glucose transport (*Slc2a1, Slc2a3*), glycolysis (*Hk1, Hk3, Pfkm*) and lower expression of genes involved in TCA cycle and OXPHOS/ETC. In contrast, AM displayed a prominent oxidative metabolic signature marked by expression of genes involved in TCA cycle and ETC. Collectively, these results suggest that both M1 and M2 macrophages mostly utilize glycolysis whereas AM primarily rely on mitochondrial oxidative phosphorylation at steady-state.

We next assessed the transcriptional profile of AM, M1 and M2 after H37Rv infection. M2 were the most responsive to *Mtb* infection with 4,136 differently expressed genes (FDR<0.05), against only 866 and 625 in M1 and AM, respectively. As expected, and despite overall differences in the magnitude of the response, genes up-regulated in response to *Mtb* infection in AM, M1 and M2 are significantly enriched among genes involved in host defense response (FDR<1.39x10^-13^, OR>2.62), cytokine mediated signaling pathway (FDR>4.09x10^-10^, OR>3.07), and the inflammatory response (FDR>1.77x10^-5^, OR>3.29). We next hypothesized that the weaker response observed in M1 as compared to M2 resulted from the fact that M2 were able to polarize towards an M1 phenotype after H37Rv infection. Consistently, we found that genes that respond exclusively to H37Rv infection in M2 and not in M1 (FDR<0.05 and >0.5 for the response to *Mtb* in M2 and M1, respectively) are significantly enriched among genes that are already differently expressed between M1 and M2 at baseline (OR=1.75; p=1.08x10^-19^). Notably, the expression of *Irg1*, a key enzyme for activation of the itaconate biosynthesis pathway linked to inflammatory macrophage phenotype (27, 28) and *Mtb* killing (28, 29), was markedly reduced in AM in comparison to either M1 (logFC=5.5, FDR=5.1x10^-12^) or M2 (logFC=3.2, FDR=1.0x10^-6^) BMDMs after H37Rv infection (**Supplementary Figure S2A**). Surprisingly, we found that upon H37Rv infection, the expression of *Irg1* drastically increased in M2 to levels expressed in M1, while AM and M1 only showed slightly upregulation. This result is consistent with the described plasticity of recruited macrophages based on the inflammatory milieu (4).

The most striking differences in response to *Mtb* infection related again to metabolic processes: in response to H37Rv infection, AM up-regulated a disproportionally large number of genes involved in cholesterol biosynthesis (enrichment OR=42.07, FDR=6.26x10^-23^), a pattern not seen in M1 or M2 macrophages (**Figure 2E**). Genes involved in cholesterol pathway were highly expressed in non-infected AM than in M1 or M2, and those differences significantly increased in AM after infection with H37Rv (**Figure 2H**). Given that *Mtb* has the unusual ability to catabolize host cholesterol as a source of carbon and energy – a function required for *Mtb* persistence in animal models (30, 31) – our data suggest that the metabolic state of AM is tailored to benefit *Mtb* survival and virulence. From a different perspective, the findings also suggest that BMDM preconditioned to either M1 or M2 differentiation are potentially capable of augmenting pulmonary host defenses to *Mtb* if they localized to the airways.

### Murine and human AM are metabolically impaired to increase glycolysis after H37Ra infection

Following the differences in cellular metabolism prior and after infection revealed by RNA-Seq, we next examined the metabolic phenotypes of AM, M1 and M2 both at resting states and during infection with a non-virulent strain of *Mtb* (H37Ra). We measured both the extracellular acidification rate (ECAR), representative of glycolysis, and oxygen consumption rate (OCR), representative of OXPHOS, of M1/M2 and AM **(** **Figures 1A–E** **)**. At baseline, OCR and ECAR were comparable between M2 and AM, but M1 had lower OCR, higher ECAR and ECAR/OCR ratio **(** **Figures 1B–D** **)**. Spare respiratory capacity (SRC), measuring the cell capacity to meet energetic demands and cell survival (32, 33), ATP production and proton efflux were significantly greater in AM compared to both M1 and M2 **(** **Figure 1E** and **Supplementary Figures 2B, C** **)**. Although AM closely resembles M2 at baseline, their unique metabolic program may contribute to their pro-survival phenotype and long half-life that can be exploited by *Mtb* for its survival (34). We then investigated whether OCR and ECAR change following infection of M1/M2 and AM with live avirulent mycobacteria (H37Ra) **(** **Figure 1F** **)**. At baseline, both M1 and M2 had higher ECAR/OCR ratios than AM, indicating a higher emphasis on glycolysis for ATP production **(** **Figure 1G** and **Supplementary Figures 2D, E** **)**. Interestingly, the SRC of AM remained high while the mitochondria of M1- and M2-BMDM operated at maximum capacity (**Figure 1H** **)**, indicating that AM consistently maintain a higher mitochondrial potential, even after infection. In addition, while all macrophages showed decreased OCR after infection, M1 and M2 displayed an inflammatory glycolytic phenotype (increased ECAR), while AM did not **(** **Figures 1I–K** **)**. We next investigated the cytokine responses of AM and M1/M2 to H37Ra infection. After infection, production of IL-12p40, IL-6 and IL-10 was significantly reduced in AM compared to M1 and M2 macrophages while the production of TNFα was intact (**Supplementary Figures S2F–I** **)**. We then evaluated the protective capacity of M1, M2 and AM. Supporting the unique AM metabolism and their anti-inflammatory phenotype, the bacterial burden within AM was significantly greater than that of M1 or M2 at both 48 and 72 hrs post-infection **(** **Figure 1L** **)**.

We finally investigated whether the responses of human AM and MDM to H37Ra infection displayed the same differences observed for murine macrophage populations. We obtained human AM *via* bronchoscopy (BAL) and generated MDM from human PBMCs to measure ECAR and OCR before and after infection with live H37Ra. Like murine AM, human AM downregulated OCR, but they did not increase ECAR **(** **Figures 1M, N** **)** after avirulent mycobacterial infection (H37Ra**).** In sharp contrast to hAM, MDM were able to increase both OXPHOS and glycolysis **(** **Figures 1O, P** **)** following H37Ra infection.

### The virulent strain of Mtb inhibits the glycolytic pathway in both AM and BMDM

**Figure 3:**
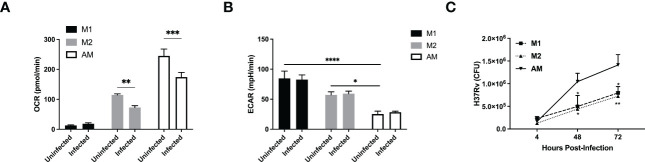
A virulent strain of Mtb inhibits the glycolytic pathway in both AM and BMDM M1/M2 bone marrow-derived and naïve alveolar macrophages were subjected to a Seahorse assay to measure cellular metabolism before and after 24hrs of infection with H37Rv at MOI of 1. **(A, B)** Quantification of basal OCR **(A)** and ECAR **(B)** in macrophages infected with H37Rv. **(C)** M1 and M2 bone marrow-derived and naïve alveolar macrophages were infected at MOI 1 with H37Rv and subjected to CFU assays. Data are given as mean ± SEM. The data are representative of 4 **(A, B)** or 3 **(C)** independent experiments. *p<0.05; **p<0.01; ***p<0.001, ****p<0.0001 determined by 2-way ANOVA followed by Tukey’s or Sidak’s multiple comparison.

Although H37Ra differentially reprogrammed the metabolism of AM or M1/M2 macrophages, this is a non-virulent strain of *Mtb* and thus, it is imperative to validate our findings with a virulent strain of *Mtb* (H37Rv). We infected the murine macrophages with the live virulent H37Rv. Similarly to H37Ra infection, both M2 and AM showed decreased OCR following infection (**Figure 3A** and **Supplementary Figure 2J**). However, while the basal ECAR were significantly higher in M1/M2 compared to AM, there was no increase in ECAR in all macrophages infected with H37Rv (**Figure 3B** and **Supplementary Figure 2K**). This suggests that compared to the avirulent strain of H37Ra, the virulent strain of H37Rv suppresses the glycolytic pathway, as also observed in a recent study (13). These data collectively indicate that the metabolic pre-programming of AM with high OCR prevent them to adopt an inflammatory phenotype (**Figures 3A, B** **)** and virulence factors from H37Rv inhibits glycolysis in macrophages.

Similar to H37Ra infection, we found that AM failed to produce cytokines, including IL-12p40, IL-6 and IL-10, but not TNFα, in response to H37Rv infection (**Supplementary Figures S2L–P** **)**. The bacterial burden within AM was significantly greater than that of M1 or M2 at both 48 and 72 hrs post-infection **(** **Figure 3C** **)**. One possible explanation is that *Mtb* has evolved to specifically target macrophage metabolism to infect and hide without triggering an inflammatory response from its host cell. Furthermore, the high levels of SRC as well as the cholesterol biosynthesis in AM might render them as a perfect host for *Mtb* replication.

### Reprogramming AM metabolism enhances their capacity to kill Mtb

**Figure 4:**
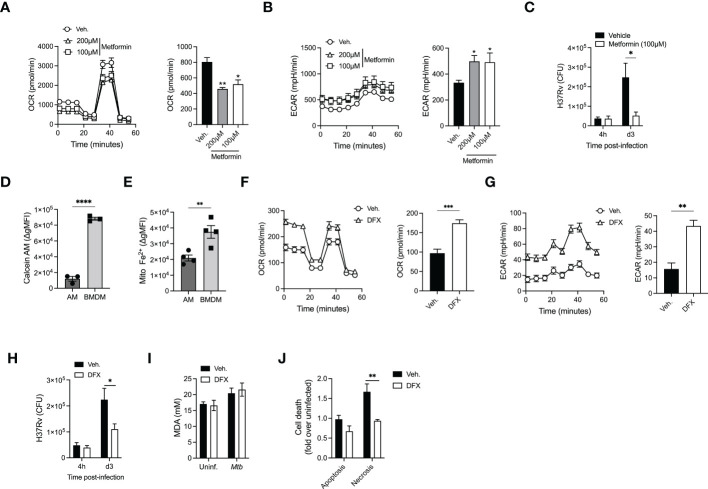
AM metabolic reprogramming enhance their killing capacities. **(A, B)** Alveolar macrophages were treated with Metformin (100µM or 200µM) before cellular metabolism, OCR **(A)** and ECAR **(B)** was evaluated using SeaHorse. **(C)** Metformin-treated AM (100µM) were infected with H37Rv (MOI=1) and bacterial loads were evaluated at different days post-infection. **(D, E)** Geometric mean fluorescence intensity (gMFI) of Calcein-AM loaded **(D)** or mitochondrial iron specific dye RPA stained **(E)** AM (SiglecF^+^CD11c^+^ from BAL) or BMDM (CD11b^+^F480^+^). **(F, G)** AM were pretreated with desferrioxamine (DFX, 100µM, 24h) and cellular metabolism- OCR **(F)** and ECAR **(G)** were evaluated. **(H)** DFX-pretreated AM (100µM, 24h) were infected with H37Rv (MOI=1) and bacterial loads were enumerated at different days post-infection. **(I)** Levels of Malondialdehyde (MDA) to measure lipid peroxidation in AM pretreated with DFX (100µM, 24h) after infection by H37Rv (MOI=10, 24h p.i). **(J)** Levels of apoptosis and necrosis in AM treated with DFX after infection by H37Rv (MOI=10, 24h p.i). Data are representative of 3-4 independent experiments. *p<0.05; **p<0.01; ***p<0.001; ****p<0.0001 determined by 1-way ANONVA followed by Dunnett’s multiple comparison, unpaired t-test or 2-way ANOVA followed by Tukey’s or Sidak’s multiple comparison test.

While our data showed that there are distinct metabolic responses in *Mtb-*infected AM and BMDM that results in promoting or limiting *Mtb* growth, respectively, we next investigated the potential link between macrophage metabolism and anti-mycobacterial responses. There is substantial evidence suggesting that the function of macrophages can be modulated *via* their metabolism (35). Therefore, we investigated whether we could pharmacologically increase glycolysis in AM and impact *Mtb* replication. We initially used Metformin (AMPK-activator), which has been shown to enhance macrophage antimycobacterial function (36). Metformin-treated AM showed a significant decrease in OCR and increase in ECAR, indicating a shift towards glycolysis, which was associated with inhibition of *Mtb* growth (**Figures 4A–C**). We next investigated the molecular mechanisms underlying AM metabolic reprogramming. A recent study suggested that AM represent an iron-replete environment promoting *Mtb* growth (37). Indeed, when we compared the iron levels in AM vs BMDM using Calcein and RPA dyes whose fluorescence is quenched in presence of iron, we found that both cytosolic labile iron pool (Calcein) and mitochondrial iron levels (RPA) were significantly higher in AM compared to BMDM (**Figures 4D, E** and **Supplementary Figures S3A, B** **)**. This is of particular importance given the critical role of iron in determining macrophage metabolism (38, 39) and response to *Mtb* infection (10). To further test the link between iron and metabolism, we treated AM with the iron chelator desferrioxamine (DFX) (38), which led to increased glycolysis (ECAR), while maintaining high levels of OCR (**Figures 4F, G** **)**. Strikingly, increased metabolism due to iron chelation in AM significantly augmented their antimycobacterial capacity (**Figure 4H**). Importantly, neither DFX nor metformin had a direct effect on the growth of *Mtb* (**Supplementary Figure S3C**) indicating that the protective impact of DFX and metformin was *via* increased glycolysis in *Mtb* infected macrophages. Whether the effects of Metformin and DFX on glycolysis are mediated by similar or different pathways will require further investigations.

We next investigated the potential contribution of iron modulation in AM cell death program, as iron has been recently shown to participate in ferroptosis during *Mtb* infection (40). Assessment of lipid peroxidation showed no difference in ferroptosis in AM infected with H37Rv (**Figure 4I**). We have previously shown that the macrophage death modality (apoptosis vs necrosis) plays an essential role in controlling the growth of *Mtb* in macrophages (16, 41, 42) and that *Mtb* hijacks RIPK3-mediated necroptosis in macrophages to promote bacterial growth (41). While no difference was observed in the levels of apoptosis, DFX treatment significantly reduced AM necrosis (**Figure 4J**). Thus, our data suggest that reprogramming AM metabolism towards glycolysis can enhance their antimycobacterial activity *via* inhibiting H37Rv-induced necrosis.

## Discussion

Despite the world-wide application of the Bacillus Calmette-Guérin (BCG) vaccination and other anti–*Mycobacterium tuberculosis* (*Mtb*) interventions, *Mtb* remains one of the most successful human pathogens. Annually, 1.6 million people die of TB, with 9 to 11.1 million new cases of active TB occurring each year (43). Epidemiological data indicate that among close household contacts of patients with active TB, approximately 50% of these individuals are intrinsically resistance to TB (44). Importantly, this resistance appears to be mainly dependent on innate immunity as these individuals remain TST negative. While this evidence suggests that pulmonary macrophages can potentially eliminate *Mtb* at the early stage after exposure, a recent study showed that adaptive immunity and, in particular, non-IFNγ producing T cells and *Mtb*-antibody specific responses may also contribute to this protection (45). An elegant study by Urdahl’s lab has demonstrated that AM are responsible for the translocation of *Mtb* from alveoli to the lung interstitium promoting *Mtb* dissemination (46). Similar to our observation, another study by the Russell lab showed that in an *in vivo* murine model of TB, AM are more permissive to *Mtb* infection due to their metabolic status, which are a nutritionally privileged niche for *Mtb* (12). However, we here demonstrated that at the steady state the level of glycolysis is significantly higher in M1 and M2 compared to AM that can be increased only in BMDM after infection with an avirulent strain (e.g. H37Ra). Importantly, we have extended our observation from murine model of macrophages to humans. Despite the expected species-specific differences in mitochondrial respiration responses (47), MDM switched towards glycolysis after Mtb infection, similar to our observations with BMDM. In sharp contrast to MDM, AM from healthy subjects are unable to energetically step up after H37Ra infection. Collectively, these murine and human results indicate that AM are metabolically wired toward mitochondrial oxidative phosphorylation. Additionally, the ability of a virulent strain (H37Rv) to suppress the glycolytic pathway in both AM and BMDM indicates that virulence factor(s) have evolved in *Mtb* to limit glycolysis, which is at least partially required for the antimicrobial function of macrophages. Absence of RD-1 region in avirulent BCG, which contain several genes essential for virulence, is the best understood genomic difference related to the attenuation of mycobacteria. However, RD-1 region is intact in H37Ra but few other genetic differences has been reported between H37Rv and H37Ra genome (48–50). Thus, a mechanistic approach to identify these virulence factors that modulate macrophages metabolism and support *Mtb* survival will significantly enhance our understanding of macrophage/*Mtb* interactions.

Our data showed that in contrast to AM, the intrinsic capacity of BMDM to increase glycolysis was associated with augmented control of H37Ra growth. Similarly, the pharmacological reprogramming of AM towards glycolysis increases their capacity to restrict H37Rv growth. However, it was unexpected that, BMDM were still able to control the growth of H37Rv more efficiently than AM, despite inhibition of glycolysis by H37Rv. We envision that there might be two possible explanations, which are not mutually exclusive. First, in the steady-state, the basal level of glycolytic pathway in BMDM is higher than AM, this might be sufficient to prevent the growth of *Mtb* during early stages of infection. Second, our observation of increased activity of the cholesterol pathway, central to growth and survival of *Mtb*, in AM only at steady-state and after H37Rv infection, provides further evidence of why *Mtb* is metabolically adapted to alveolar macrophages (51, 52). Thus, the AM/cholesterol pathway can be targeted for host-directed therapy using lipid/cholesterol-lowering drugs such as statins, which have already been proposed as a potential anti-TB treatment by enhancing macrophages antimycobacterial capacity (53–55). Whether statins directly regulate macrophage metabolism will require further investigation. In addition, our data indicate a critical role for iron in shaping the metabolic profile of AM, which have increased iron levels compared to BMDM. This is in agreement with the observations that *Mtb* relies less on siderophores to capture iron within AM, suggesting an iron-replete environment (37). Using a well-known iron chelator, desferrioxamine, we were able to reprogram AM metabolism towards glycolysis to enhance their antimycobacterial activity by specifically limiting *Mtb*-induced necrosis. This is to our knowledge, the first report suggesting a link between AM metabolism, iron and cell death following *Mtb* infection. While we have recently shown that *Mtb* induces RIPK3-mediated necroptosis in myeloid progenitors *via* modulation of iron (10), the potential involvement of a glycolysis/RIPK3/iron axis in induction of necrosis in *Mtb*-infected macrophages requires further scrutiny. Interestingly, we also found that both murine and human AM are metabolically wired toward mitochondrial OXPHOS in comparison to bone marrow/monocyte-derived macrophages. These data collectively add to the notion that AM are metabolically locked in their basal state due to high iron content and unable to modulate energy pathways mainly due to their unique ontogeny.

While our model is limited as it does not take into account the effects of the pulmonary microenvironment that can influence macrophage metabolism to generate a broader metabolic spectrum of macrophages (14), our data are in accordance with a recent *in vivo* study of murine model of tuberculosis (12), and are in line with the notion that a robust energetic response supports macrophage host defense (15), but viable *Mtb* infection can diminish the protective function of macrophages (13, 56). Thus, although the unique intrinsic metabolic programming of AM to avoid excessive responses in the lung may render these cells a perfect target for *Mtb*, the capacity to recruit BMDM to the airways may represent one component of protective recall responses to *Mtb* infection. Our results furthermore demonstrate the metabolic plasticity of monocyte-derived macrophage subsets, in response to inflammatory cues. Though the energetics of M2 macrophages at steady-state are more like AM, following H37Rv infection M2 macrophages showed the greatest metabolic plasticity, becoming metabolically indistinguishable from M1 macrophages (**Figures 1I, J** **)**. Importantly, the increase of glycolysis in M2 after H37Rv infection was associated with an increase in *Irg1* expression, a key enzyme in itaconate synthesis, which has been linked to macrophage antimycobacterial capacity (28, 29). Thus, the ability of M2 macrophages to control *Mtb* growth might be linked to itaconate synthesis, a phenotype that was not observed in AM. This may also provide a clue as to how recruited macrophages, despite being ontogenically distinct, can occupy tissue-resident macrophage niches, during loss of resident populations either *via* aging or severe inflammatory insults (53, 57, 58).

We have previously shown that intravenous BCG administration provides access to the BM allowing for the reprogramming of HSCs in a IFNγ-dependent manner to generate monocytes/macrophages with greater pulmonary localization and protective capacity in response to subsequent *Mtb* infection (5). Because IFNγ has also been shown to induce trained immunity in AM *via* metabolic reprogramming (59), it is tempting to speculate that intravenous BCG vaccination may also induce trained immunity in AM by increasing their metabolism towards glycolysis. In contrast to BCG, we have recently demonstrated that following pulmonary *Mtb* infection, bacteria rapidly access the bone marrow and reprogram HSCs in an iron/type 1 IFN dependent manner to generate permissive monocytes/macrophages that favor the growth of *Mtb* (10). Thus, while *Mtb* modulate local AM metabolism for its initial replication in the lung, it also accesses the bone marrow (an immune privileged site) to reprogram immune progenitor cells and compromise immunity. Further studies of protective BMDM signatures that can be localized to the lung will provide a novel approach for developing optimal treatments and vaccination strategies against TB.

## Data availability statement

The original contributions presented in the study are included in the article/**Supplementary Material**, further inquiries can be directed to the corresponding author/s. The original raw RNA-sequencing data are publicly available in the GEO repository under accession number: GSE219072.

## Ethics statement

The studies involving human participants were reviewed and approved by MUHC Research Ethics Board, study number MP-CUSM-15-406. The patients/participants provided their written informed consent to participate in this study. The animal study was reviewed and approved by Animal Research Ethics Board of McGill University, project ID: 5860.

## Author contributions

Investigations: LM, EP, NK, JS, EK, JD, AG, MO. Methodology: LM, EP, NK, JS, MO, ES, CK, RJ, LB, MD. Project administration: ES, LB, MD. Funding acquisition: LB, MD. All authors: writing, reviewing and editing. MD conceived the project. All authors contributed to the article and approved the submitted version.

## Supplementary material

Supplementary Table 1 Differential expression statistics in RNA-Seq data from murine macrophages.

Supplementary Table 2 Gene ontology enrichment analyses.

Supplementary Figure 1
*In vitro* model of M1/M2 and alveolar macrophages **(A)** Histogram depicting the expression of CD206 as measured by flow cytometry following 24hr polarization. Quantification of median fluorescence intensity (MedFI) of CD206 expression following 24hr polarization. **(B)** Histogram depicting the expression of CD86 as measured by flow cytometry following 24hr polarization. Quantification of MedFI of CD86 expression following 24hr polarization. Three sample replicates were individually analyzed and the data are given as mean ± SEM ** p<0.01; **** p<0.0001 (1-way ANOVA followed by Tukey comparison).

Supplementary Figure 2 Alveolar macrophages fail to produce critical anti-mycobacterial mediators following infection. **(A)** *Irg1* expression pattern in alveolar vs M1 and M2 macrophages at baseline (filled symbols) or in response to H37Rv infection (hollow symbols). **(B, C)** Quantification of ATP production **(B)** and proton efflux **(C)** in AM or M1/M2 BMDM at steady state. **(D, E)** M1/M2 BMDM or AM were infected with Mtb H37Ra (MOI2.5) before measuring ATP production **(D)** or proton efflux **(E)** 24 hours post-infection. **(F–I)** M1 and M2 bone marrow-derived and naive alveolar macrophages were infected at MOI 2.5 with H37Ra and cytokines release by infected macrophages was measured by ELISA. **(J, K)** OCR and ECAR curves following infection by H37Rv (24 hours, MOI=1). **(L–O)** M1 and M2 bone marrow-derived and naive alveolar macrophages were infected at MOI 1 with H37Rv and cytokines release by infected macrophages was measured by ELISA. Data are given as mean ± SEM. The results are representative of 2 (B-I) or 3 **(J–O)** independent experiments. * p<0.05; ** p<0.01; *** p<0.001; **** p<0.0001 compared with AM (2-way ANOVA followed by Dunnett’s comparison).

Supplementary Figure 3 Iron chelation has no effect on *Mtb* growth. **(A, B)** Representative histograms of calcein-AM **(A)** or RPA **(B)** stained AM or BMDM. **(C)** Growth of *Mtb* H37Rv in presence of 100 or 200µM of DFX or Metformin.
