# Supplementary figures and images for: Human alveolar macrophage metabolism is compromised during Mycobacterium tuberculosis infection
Source: Front Immunol. 2023 Jan 26;13:1044592. doi: 10.3389/fimmu.2022.1044592 (PMC9910175; doi:10.3389/fimmu.2022.1044592)

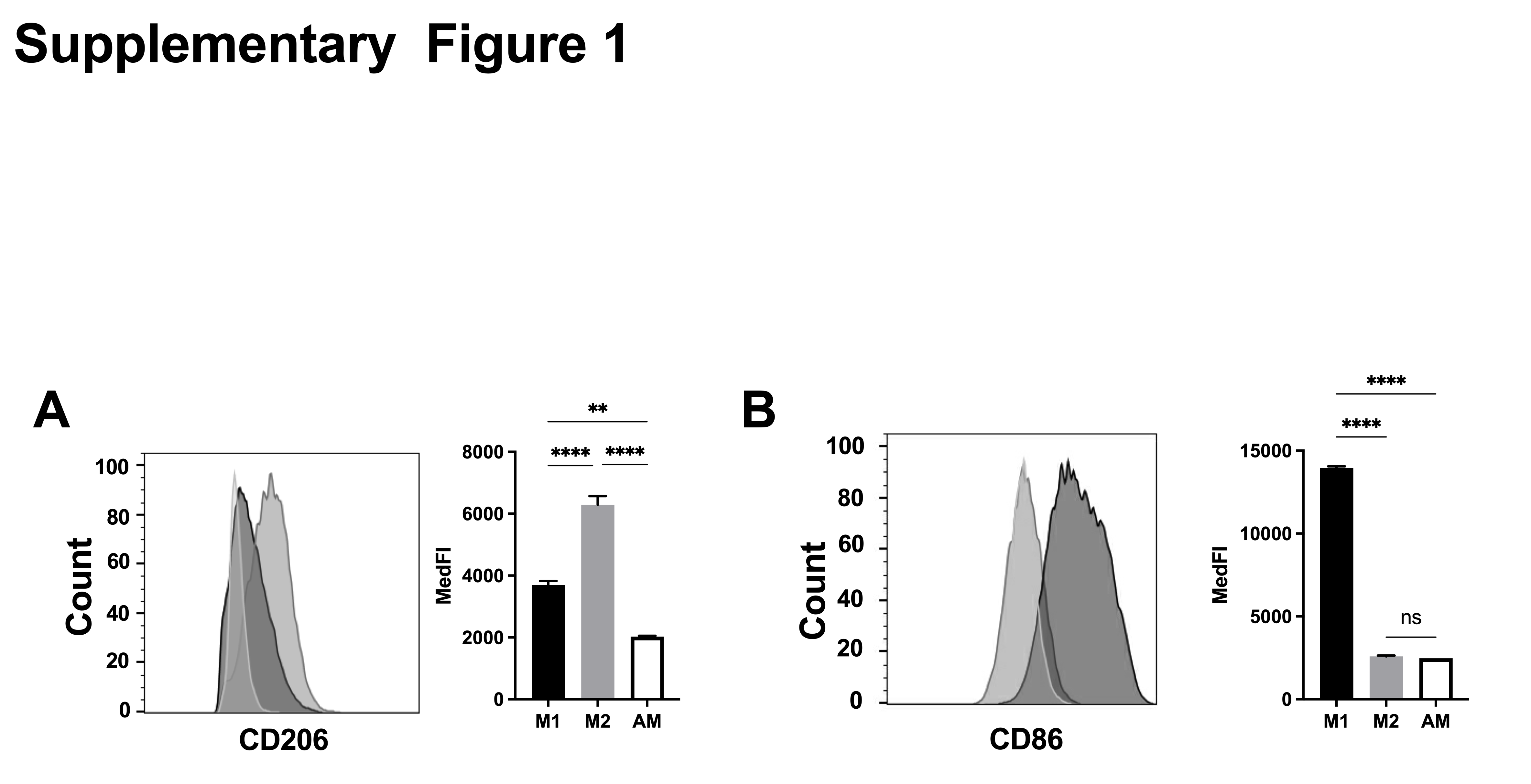

Supplement: Supplementary Figure 1 — In vitro model of M1/M2 and alveolar macrophages (A) Histogram depicting the expression of CD206 as measured by flow cytometry following 24hr polarization. Quantification of median fluorescence intensity (MedFI) of CD206 expression following 24hr polarization. (B) Histogram depicting the expression of CD86 as measured by flow cytometry following 24hr polarization. Quantification of MedFI of CD86 expression following 24hr polarization. Three sample replicates were individually analyzed and the data are given as mean ± SEM ** p<0.01; **** p<0.0001 (1-way ANOVA followed by Tukey comparison). [file Image_1.tiff]

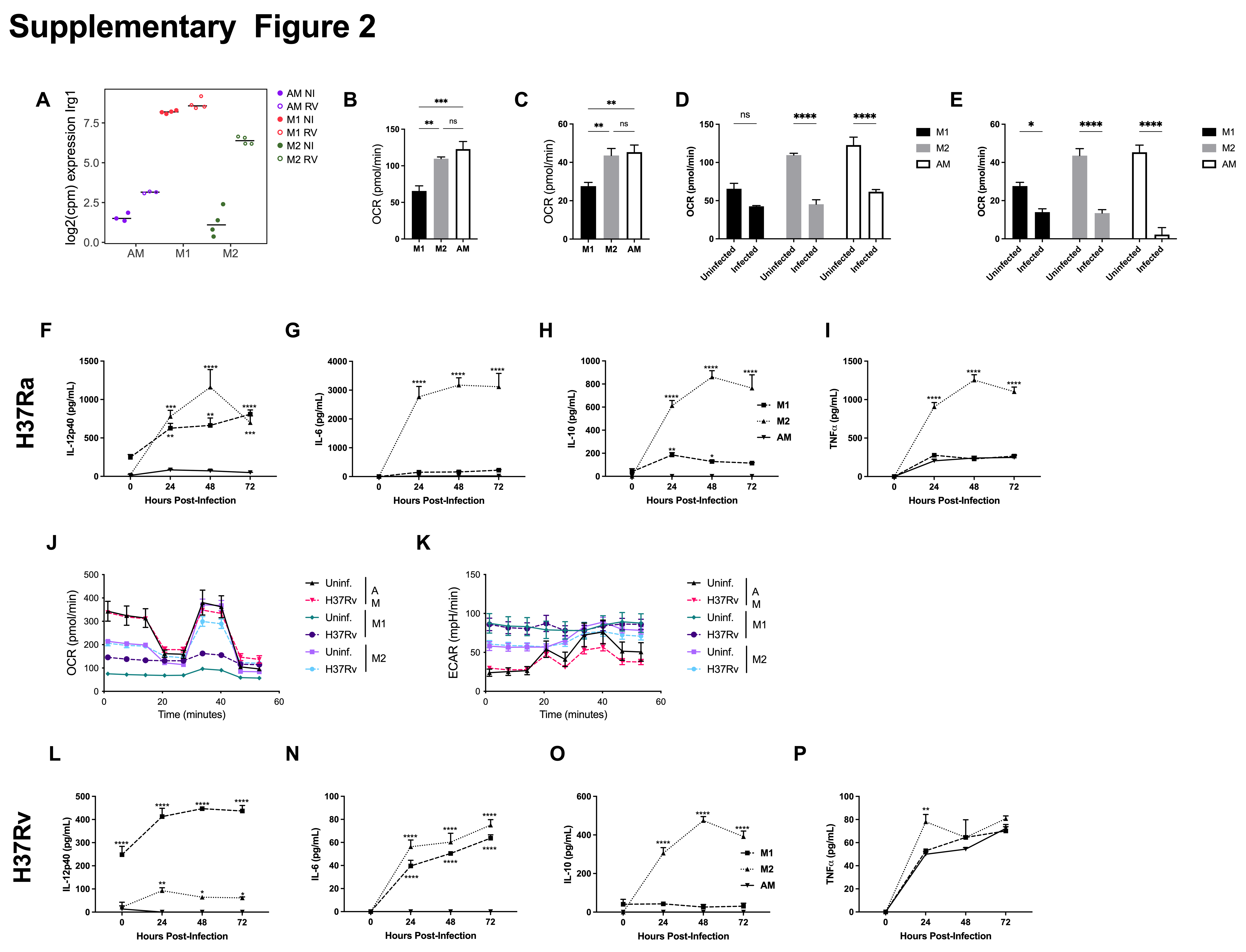

Supplement: Supplementary Figure 2 — Alveolar macrophages fail to produce critical anti-mycobacterial mediators following infection. (A) Irg1 expression pattern in alveolar vs M1 and M2 macrophages at baseline (filled symbols) or in response to H37Rv infection (hollow symbols). (B, C) Quantification of ATP production (B) and proton efflux (C) in AM or M1/M2 BMDM at steady state. (D, E) M1/M2 BMDM or AM were infected with Mtb H37Ra (MOI2.5) before measuring ATP production (D) or proton efflux (E) 24 hours post-infection. (F–I) M1 and M2 bone marrow-derived and naive alveolar macrophages were infected at MOI 2.5 with H37Ra and cytokines release by infected macrophages was measured by ELISA. (J, K) OCR and ECAR curves following infection by H37Rv (24 hours, MOI=1). (L–O) M1 and M2 bone marrow-derived and naive alveolar macrophages were infected at MOI 1 with H37Rv and cytokines release by infected macrophages was measured by ELISA. Data are given as mean ± SEM. The results are representative of 2 (B-I) or 3 (J–O) independent experiments. * p<0.05; ** p<0.01; *** p<0.001; **** p<0.0001 compared with AM (2-way ANOVA followed by Dunnett’s comparison). [file Image_2.tiff]

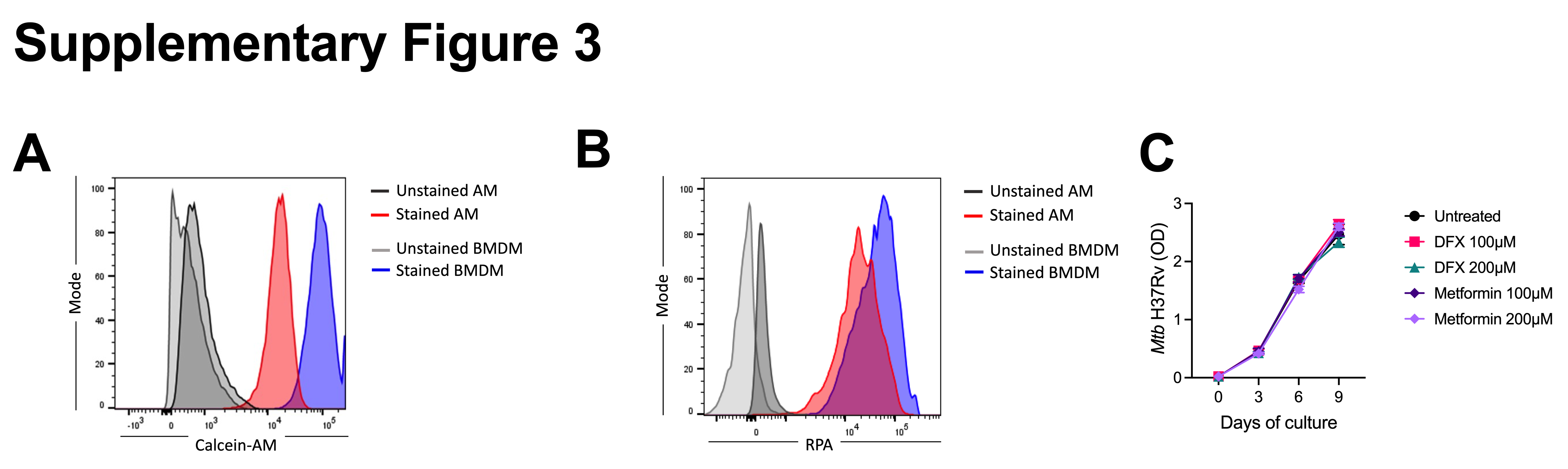

Supplement: Supplementary Figure 3 — Iron chelation has no effect on Mtb growth. (A, B) Representative histograms of calcein-AM (A) or RPA (B) stained AM or BMDM. (C) Growth of Mtb H37Rv in presence of 100 or 200µM of DFX or Metformin. [file Image_3.tiff]
